# IL-27 Receptor Signaling on T cells Augments GVHD Severity through Enhancing Th1 Responses

**Published:** 2018-06-07

**Authors:** David Bastian, Yuejun Liu, Yongxia Wu, Steven Schutt, Hung D. Nguyen, Anusara Daenthanasanmak, M.Hanief Sofi, Mengmeng Zhang, Supinya Iamsuwat, Xue-Zhong Yu

**Affiliations:** 1 Department of Microbiology and Immunology, Medical University of South Carolina, USA; 2 Institute of Blood and Marrow Transplantation, Department of Hematology, The First Affiliated Hospital of Soochow University, China; 3 Department of Medicine, Medical University of South Carolina, USA

## Abstract

IL-27 is a heterodimeric cytokine comprised of IL-27p28 and EBI3. As a relatively new member of the IL-12 family, the biological mechanisms associated with the role of IL-27 in the immune response are ambiguous, displaying both proinflammatory and suppressive functions that seem to be dependent on the disease model. A recent report demonstrates that pharmacological blockade of IL-27p28 alleviates graft-versus-host disease (GVHD) in mice. However, the specific role of the IL-27Rα/gp130 signaling complex that forms the IL-27 receptor (IL-27R) on T cells has not been well characterized in the context of allogeneic hematopoietic stem cell transplantation (allo-HCT). Here, we demonstrate that IL-27Rα expression on T cells exacerbates GVHD after allo-HCT, which was consistent across 3 different MHC- mismatched murine models of allo-HCT. Expression of IL-27Rα on T cells was required for acquisition of optimal Th1 effector function and subsequent inhibition of Th2 and T regulatory subsets after allo-HCT. Furthermore, administration of IL-27significantly increased mortality after allo-HCT; suggesting that the suppressive functions linked to IL-27 in T cell responses may be relatively modest in this model. Hence, IL-27Rα signaling on T cells promotes the development of GVHD.

## INTRODUCTION

Interleukin-27 (IL-27) is a heterodimeric cytokine belonging to the IL-12 family. IL-27 is comprised of an IL-27p28α-chain and an EBI3β- chain and is the only member of the family that is not secreted as a functional dimer [1,2]. As such, the receptor for IL-27is also heterodimeric, and is composed of a unique IL-27 receptor (IL-27R) component, or WSX-1, that forms a complex with gp130 to transduce signaling [3]. Activated dendritic cells (DCs) and monocytes serve as the primary source of p28 and EBI3 [1]. IL-27Rα is expressed in low levels on naïve T cells, but is upregulated on effector and memory T cells [4]. The biological mechanisms associated with the role of IL-27 in the immune response are ambiguous, displaying both proinflammatory and suppressive functions that seem to be dependent on the disease model.

Allogeneic hematopoietic stem cell transplantation (allo-HCT) is an effective means by which to treat a wide variety of diseases resulting from dysfunctional hematopoiesis; ranging from certain immune deficiencies to severe blood diseases and cancers [5]. However, the development of graft-versus-host disease (GVHD) remains the major cause of morbidity and mortality after allo-HCT. Acute GVHD (aGVHD) generally occurs in the first 100 days post allo-HCT and is a result of donor T cell recognition of genetically disparate antigens presented by antigen presenting cells (APCs), which subsequently leads to activation of both the innate and adaptive immune responses against host epithelial tissues; namely the skin, lung, liver and gastrointestinal tract (GI tract) [6].

A recent report demonstrates that pharmacological blockade of IL-27p28 alleviates GVHD in mice [7]. However, the specific role of the IL-27R/gp130 signaling complex that forms the IL-27 receptor on T cells during GVHD development is still unclear. Hence, we evaluated the role of IL-27R signaling in T cell responses to alloantigen across multiple MHC- mismatched models of allo-HCT, and found that IL-27Rα expression promotes T cell pathogenicity attributable to augmented Th1 effector function.

## MATERIALS AND METHODS

### Mice:

C57BL/6 (B6; H-2^b^), BALB/c (H-2^d^), B6-Ly5.2 (H-2^b^), B6D2F1 (B6 × DBA2) F1 (H-2^b/d^) were purchased from NCI. IL-27R KO and BALB. B mice were purchased from Jackson Labs. All animals were housed in specific pathogen-free conditions in the America Association for Laboratory Animal Care-accredited Animal Resource Center at the Medical University of South Carolina (MUSC). The Institutional Animal Care and Use Committee of MUSC approved all work.

### GVHD models:

Using an X-RAD 320 irradiator, lethally irradiated recipient BALB/c (650cGy), BALB. B (900cGy) or B6D2F1 (1200cGy) recipients were transplanted with 5×10^6^T cell depleted bone marrow (TCD-BM) alone or TCD-BM plus 1-3×10^6^WT or IL-27R KO T cells and monitored for survival and body weight loss as previously described [8-11]. T cells were purified from pooled spleen and lymph node cells by negative selection to remove non-T cells including B cells, natural killer (NK) cells, DCs, macrophages, granulocytes, and erythroid cells. Briefly, non-T cells were magnetically labeled with biotin-conjugated Abs against CD45R (B220), CD49b (DX5), CD11b (Mac-1), and Ter-119, followed by anti-biotin MicroBeads (Miltenyi Biotech, Auburn, CA). Isolation of T cells was achieved by negative selection. Bone marrow (BM) was harvested from tibia and femurs, and T cells were depleted through complement lysis of Thy1.2+ cells.

For experiments involving adenoviral production of IL-27, mice were injected intramuscularly with 2×10^11^ DRP of vectors 7 days prior to BMT with either vector control or IL-27AAV [12].

### Flow cytometry and intracellular cytokine staining:

Mononuclear cells were isolated from recipient spleen or liver as previously described and stained for surface markers and intracellular cytokines using standard flow cytometric protocols [10,11]. Stained cells were analyzed using FACSDiva software, LSR II (BD Biosciences, San Jose, CA), and FlowJo (Tree Star, Ashland, OR).The following Abs were used for cell-surface staining: anti-CD4–V450, -APC, and -PEcy7 (BD Biosciences), anti-CD8-PEcy5, -APCcy7 and -AF700 (BD Biosciences,); anti–CD45.1-FITC, - and -APC (BD Biosciences). Intracellular staining was carried out using anti-IFN-γ-PE or Per-cp 5.5 (XMG1.2; BD Biosciences), anti–IL-4–PE (11B11; BD Pharmingen), anti–IL-5–PE (TRFK5; BD Pharmingen), anti-Foxp3–PE (FJK-16s; eBioscience).

### Statistics:

For comparison of recipient survival among groups in GVHD experiments, the log-rank test was used to determine statistical significance. To compare body weight changes and cytokine levels, a Student t test was performed.

## RESULTS

### IL-27R is required for T cells to induce GVHD

**Figure 1.**
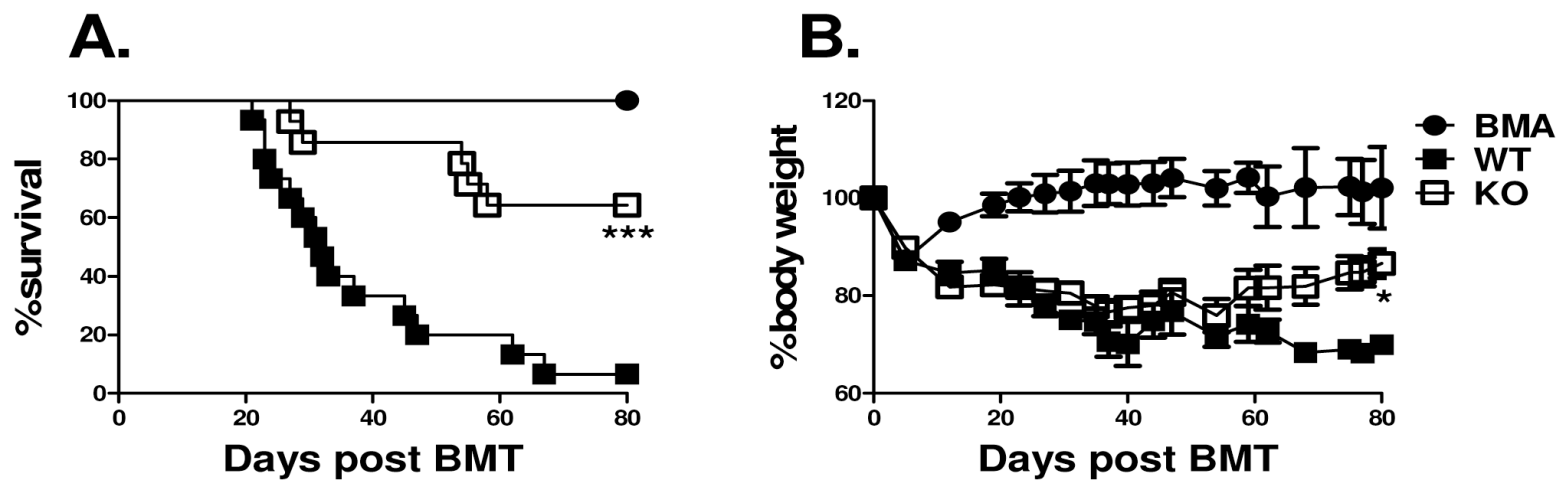
Role of IL-27R on T cells in miHA-mismatched GVHD. Lethally irradiated BALB.B mice were transplanted with 5×10^6^ TCD-BM alone(BMA)or plus 3×10^6^ purified T cells from WT B6 or IL-27R KO mice. Percentages of recipient survival (A) and body weight loss (B) are shown. Data shown is pooled from 2 replicate experiments with a total of 4 BALB.B mice that received TCD-BM alone and 10 BALB.B mice per group that received T cells. * p<.05; *** p<.001

Given the recent findings that IL-27p28 exacerbates graft-versus-host disease (GVHD), we hypothesized that targeting the alpha receptor subunit of the IL-27 receptor (IL-27Rα) specifically on T cells would result in a reduction in GVHD severity after allogeneic bone marrow transplantation (allo-BMT). In order to decipher the role of IL-27Rα on T cells, we initially tested the ability of IL-27R deficient T cells to cause GVHD in a MHC-matched but minor histocompatibility antigen (miHA) mismatched murine BMT model, C57BL/6 to BALB.B. Recipients that received T cells deficient for IL-27Rα developed less severe GVHD, as shown by a significantly higher survival percentage across experiments compared to WT controls (Figure 1A); which correlated with significantly improved body weight maintenance among groups that received IL-27Rα deficient T cells (Figure 1**B**). Hence, T cells deficient for IL-27Rαhave a compromised ability to induce GVHD in a MHC-matched model of allo-BMT.

### IL-27R signaling augments Th1 responses

**Figure 2.**
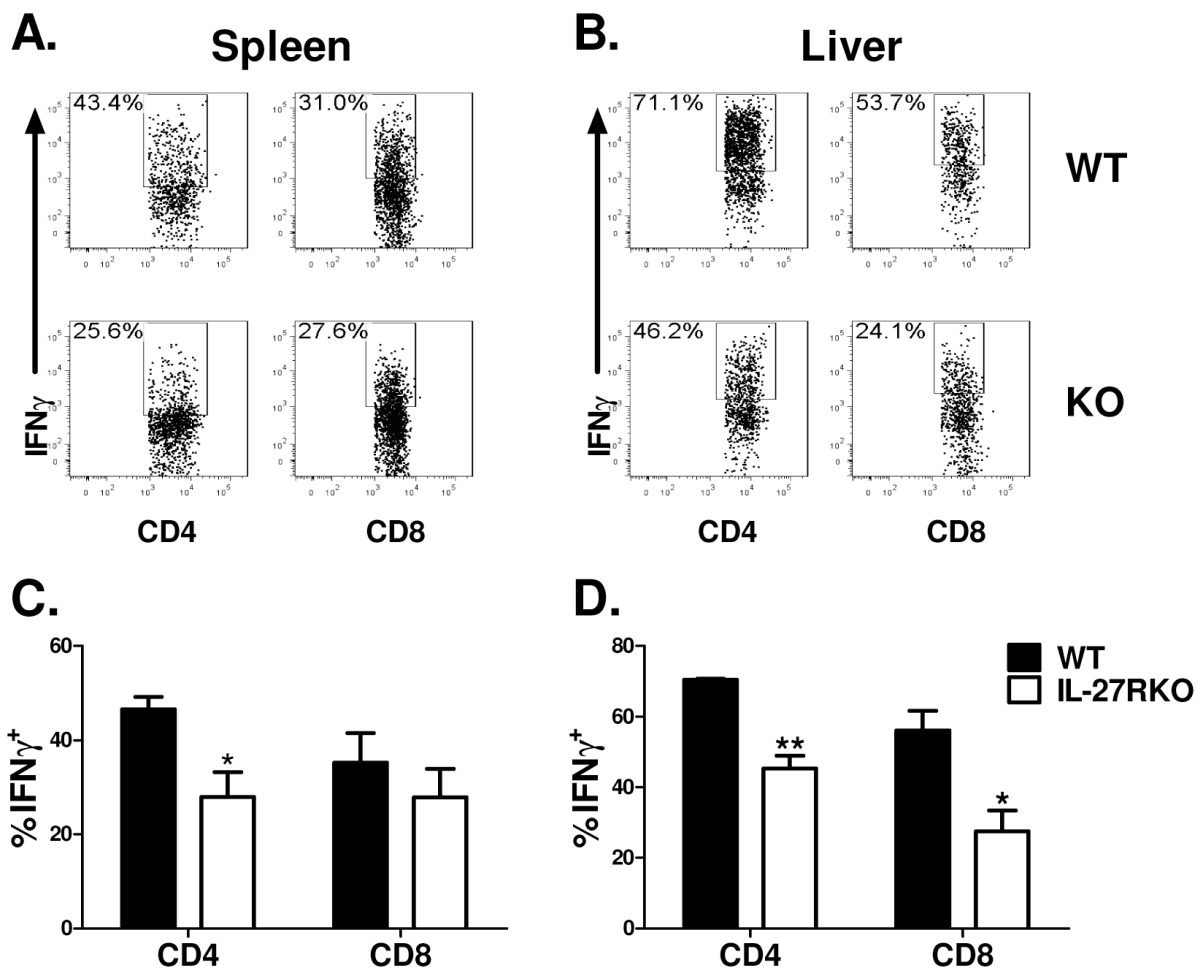
Effect of IL-27R on donor T cells in response to miHA. Lethally irradiated BALB.B mice were transplanted with 5×10^6^ Ly5.1^+^ TCD-BM alone or plus 3×10^6^ purified T cells (Ly5.2^+^) from WT B6 or IL-27R KO mice. Twenty-one days post-BMT, recipient spleens and livers were collected and mononuclear cells were isolated and stained for surface and intracellular markers for analysis by flow cytometry. Flow cytometry data is depicted for 1 representative mouse per group on IFNγ^+^ expression among gated Ly5.1^−^ CD4^+^ or CD8^+^ cells in the spleen (A) and liver (B). Average percentages of CD4^+^ or CD8^+^ T cells positive for IFNγ among gated Ly5.1^−^ CD4^+^ or CD8^+^ cells in the spleen (C) and liver (D) are shown. Data from 1 representative experiment with 4 mice per group is shown. The total number of mice analyzed in these experiments in the WT group was 3 and the total number group in the IL-27R KO group was 4. * p<.05; ** p<.01

IL-27 was initially reported to be involved in Th1 differentiation [1]. Therefore, we hypothesized that a decrease in IFNγ production by T cells might be responsible for the alleviated GVHD burden seen in the MHC-matched BMT model (Figure 1A,1B). Consistent with this hypothesis, we found a significant decrease in the percentage of IFNγ+ T cells in the spleen (Figure 2A,2C) and liver (Figure 2B,2D) of cohorts that received IL-27RαKO T cells 21 days’ post BMT. This data demonstrates that IL-27Rα plays a role in T cell pathogenesis during GVHD development, and that this pathogenicity is, at least in part, attributable to an IL-27Rα-dependent Th1 effector response.

### IL-27R is required for optimal T cell pathogenicity across multiple BMT models

**Figure 3.**
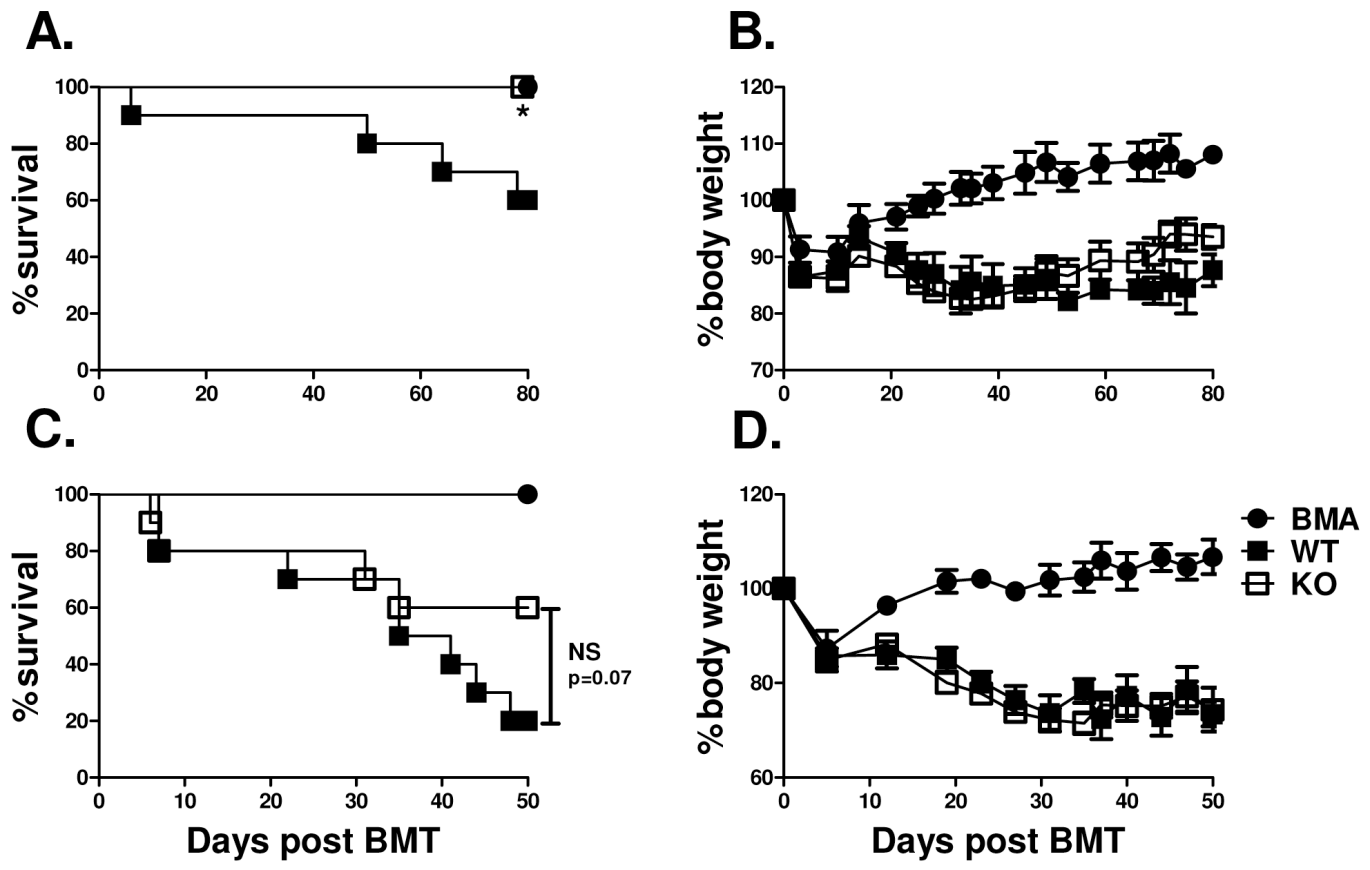
Role of IL-27R on T cells in haplo-identical or fully mismatched models of GVHD. Lethally irradiated B6D2F1 (A, B) or BALB/c (C, D) mice were transplanted with 5×10^6^ TCD-BM alone or plus 3×10^6^ (B6D2F1) or 1×10^6^ (BALB/c) purified T cells from WT B6 or IL-27R KO mice. Percentages of recipient survival (A, C) and body weight loss (B, D) are shown. Data shown is pooled from 2 replicate experiments with a total of 4 BALB/c or B6D2F1 mice that received TCD-BM alone and 10 BALB/c or B6D2F1 mice per group that received T cells. * p<.05

Since we observed that CD4+ T helper cell differentiation was significantly altered in IL-27R deficient T cells after allo-BMT in the B6 to BALB.B model, we hypothesized that a similar reduction in GVHD may also hold true in additional models of allo-BMT. To address this hypothesis, we used both the B6 to B6D2F1 (Figure 3A,3B) haplo identical BMT model as well the B6 to BALB/c (Figure 3C,3D) full MHC-mismatched model. In both models, we found an increase in survival percentage (Figure 3A,3C) among recipients of IL-27R KO T cells compared to WT controls after allo-BMT, albeit not statistically significant in BALB/c recipients. This data confirms that IL-27R expression on T cells plays a pathogenic role in GVHD development across multiple murine BMT models.

### IL-27R expression on T cells inhibits differentiation toward Th2 and T regulatory subsets after allo-BMT

**Figure 4.**
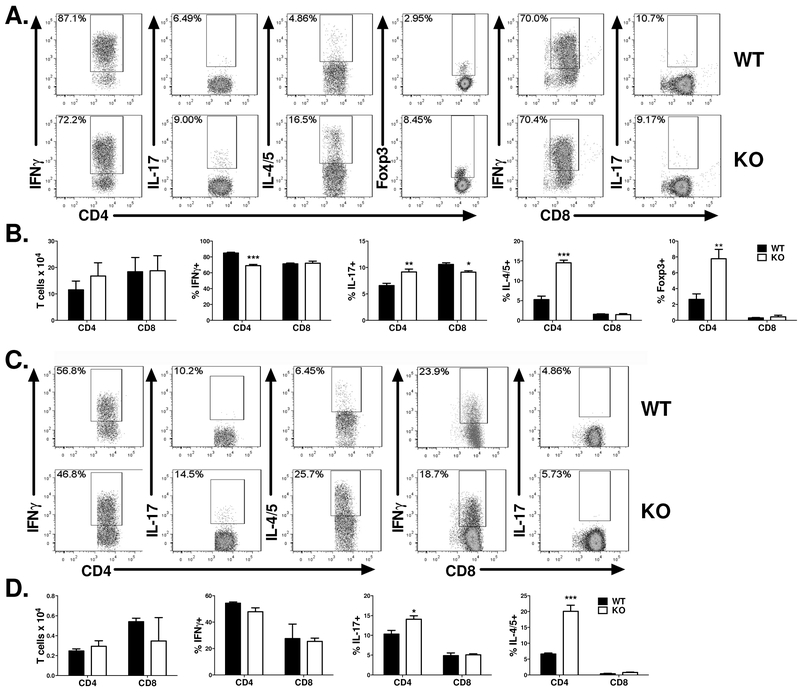
Effect of IL-27R on donor T cells during GVHD development. Lethally irradiated BALB/c mice were transplanted with 5×10^6^ Ly5.1^+^ TCD-BM alone or plus 1×10^6^ purified T cells (Ly5.2^+^) from WT B6 or IL-27R KO mice. 21 days post-BMT, recipient spleens and livers were collected and mononuclear cells were isolated and stained for surface and intracellular markers for analysis by flow cytometry. Flow cytometry data is depicted for 1 representative mouse per group for percentages of IFNγ^+^, IL-4/5^+^, IL-17^+^, or Foxp3^+^ (Tregs) among gated H2Kb^+^ Ly5.1^−^ CD4^+^ or CD8^+^ cells in the spleen (A) and liver (C). Average percentages of CD4+ or CD8+ T cells positive for IFNγ, IL-4/5, IL-17, or Foxp3 among gated H2Kb^+^ Ly5.1^−^ CD4^+^ or CD8^+^ cells in the spleen (B) and liver (D) are shown. Data from 1 out of 2 replicate experiments with 4 mice per group are shown. The total number of mice analyzed in these experiments in the WT group was 8 and the total number group in the IL-27R KO group was 6. * p<.05; ** p<.01, *** p<.001

Our previous results indicate that IL-27R expression on T cells can promote GVHD after allo-BMT and that this is attributable to an increased Th1 response. In order to corroborate this mechanism by which IL-27R on T cells influences the development of GVHD, we analyzed T cell proliferation and differentiation in the spleen and liver of BALB/c recipients 21 days’ post BMT (Figure 4). In the spleen, we saw a significant decrease in the percentage of IFNγ produced by CD4+ T cells in recipients that received IL-27R KO T cells compared to WT controls. Additionally, IL-27R KO CD4+ T cells isolated from the spleen produced a significantly higher percentage of IL-4/5 and had a significantly increased percentage of Foxp3 expression (Figure 4A,4B). Consistently, recipients of IL-27R KO T cells had a significantly higher percentage of CD4+IL-4/5+ T cells in the liver (Figure 4C,4D). These data indicate that T cells deficient for IL-27R are skewed away from Th1 differentiation and instead differentiate intoTh2 and T regulatory subsets, a pattern which would be manifested by reduced GVHD. However, we also noted significant increases, albeit among few cells, in IL-17 production by IL-27R KO CD4+ T cells in both the spleen (Figure 4 A,4B) and the liver (Figure 4C,4D).

### Administration of IL-27 exacerbates GVHD

**Figure 5.**
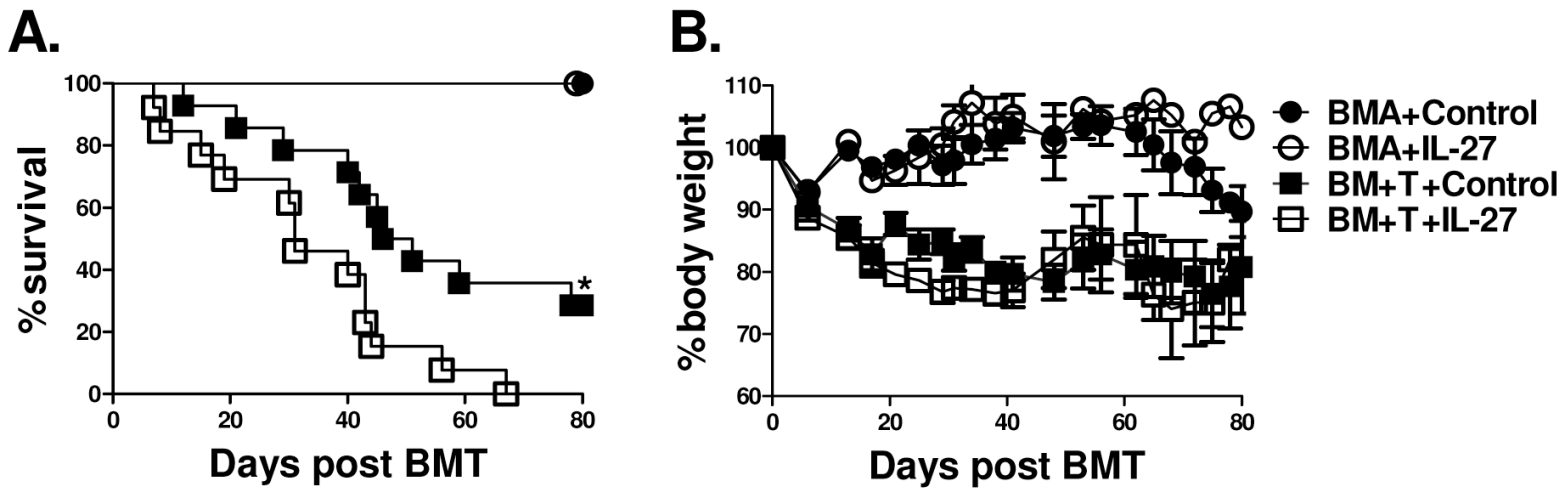
Effect of IL-27 administration in GVHD. Lethally irradiated BALB/c mice were transplanted with 5×10^6^ TCD-BM alone or plus 1×10^6^ purified T cells from WT B6 or IL-27R KO mice. Percentages of recipient survival (A) and body weight loss (B) are shown. Mice were injected intramuscularly with 2×10^11^ DRP of vectors 7 days prior to BMT with either vector control or IL-27AAV. Data shown is pooled from 3 replicate experiments with a total of 6 BALB/c mice that received TCD-BM alone with vector control or IL-27AAV and 14 BALB/c mice per group that received T cells plus vector control and 13 BALB/c mice per group that received T cells plus IL-27AAV. * p<.05

Our results specifically demonstrate that IL-27R expression on T cells augments GVHD. Taken together with previous literature implicating IL-27p28 as a proinflammatory mediator of GVHD development, we sought to delineate whether the cytokine IL-27 could augment T cell mediated GVHD. To address this question, we injected either an adenoviral vector expressing the IL-27 heterodimer (IL-27AAV) or empty vector into BALB/c recipients 7 days prior to BMT. Lethally irradiated recipient mice were then transplanted with B6 T cells and TCD-BM as described in figure 1 and monitored for survival (Figure 5A) and body weight loss (Figure 5B). Recipient mice that were treated with IL-27AAV had significantly higher mortality than those that received empty vector (Figure 5A); providing further support for the notion that IL-27 signaling on T cells promotes the development of GVHD.

## DISCUSSION

In the context of autoimmunity, IL-27 has been implicated in both pro- and anti-inflammatory responses. Taken together with the recent report advocating for pharmacological blockade of p28 as a potential therapy for GVHD, our results unambiguously demonstrate that IL-27 signaling on T cells exacerbates GVHD after allo-HCT.

In this study, we observed a consistent increase in GVHD severity; not only in a MHC-matched model of allo-BMT, but also in MHC complete-mismatched as well MHC haploidentical BMT model in cohorts that received T cells expressing IL-27R. Mechanistically, we observed that Th1 responses were augmented in cohorts that received IL-27R competent T cells, while Th2 and Treg differentiation were significantly decreased, consistent with previous reports. Of note, the observed increase in IL-4/5 production by donor T cells was quite dramatic. This is supported by previous studies which implicate IL-27 signaling as a critical regulator of T-bet and IL-12Rβ2 expression in T cells, and further demonstrate that these Th1-promoting factors consequently suppress the master Th2 transcription factor, GATA3 [13].

Our results indicate that IL-17 production was significantly decreased in mice that receivedIL-27R expressing T cells, which could be a potential explanation for why we did not observe a significant difference in body weight maintenance throughout our experiments. The increase in IL-17 production by IL-27R deficient T cells could be potentially explained by the significant reduction in IFNγ production, which has been reported to negatively regulate Th17 differentiation and, hence, IL-17 production [14]. Rather, the decreased function of Th1 cells, which hypothetically would alleviate GVHD, was offset by an increased Th17 response, resulting in Th17-mediated pathology and subsequently no difference in weight maintenance could be observed. This is supported by reports demonstrating IL-27a signaling negatively regulates Th17 differentiation using other models of autoimmunity [15,16].

In addition to demonstrating that IL-27 signaling on T cells promotes the development of GVHD, we investigated the effect of IL-27 administration after allo-BMT. In these experiments, we observed that excess IL-27 significantly increased GVHD severity. Hence, our results address the role of IL-27 in GVHD in 2 different ways, and further substantiate the claim that IL-27 signaling exacerbates GVHD. In conclusion, we provide additional evidence that IL-27 signaling is detrimental in GVHD and advocate that this pathway could be a potentially efficacious therapeutic target in clinical settings.
